# Formation of a TBX20-CASZ1 protein complex is protective against dilated cardiomyopathy and critical for cardiac homeostasis

**DOI:** 10.1371/journal.pgen.1007011

**Published:** 2017-09-25

**Authors:** Leslie Kennedy, Erin Kaltenbrun, Todd M. Greco, Brenda Temple, Laura E. Herring, Ileana M. Cristea, Frank L. Conlon

**Affiliations:** 1 University of North Carolina McAllister Heart Institute, UNC-Chapel Hill, Chapel Hill, NC, United States of America; 2 Integrative Program for Biological & Genome Sciences, UNC-Chapel Hill, Chapel Hill, NC, United States of America; 3 Department of Genetics, UNC-Chapel Hill, Chapel Hill, NC, United States of America; 4 Department of Biology, UNC-Chapel Hill, Chapel Hill, NC, United States of America; 5 Department of Molecular Biology, Princeton University, Princeton, NJ, United States of America; 6 R.L. Juliano Structural Bioinformatics Core, Department of Biochemistry and Biophysics, UNC-Chapel Hill, Chapel Hill, NC, United States of America; 7 UNC Proteomics Core Facility, UNC-Chapel Hill, Chapel Hill, NC, United States of America; 8 Department of Pharmacology, UNC-Chapel Hill, Chapel Hill, NC, United States of America; Children's Hospital Boston and Harvard Medical School, UNITED STATES

## Abstract

By the age of 40, one in five adults without symptoms of cardiovascular disease are at risk for developing congestive heart failure. Within this population, dilated cardiomyopathy (DCM) remains one of the leading causes of disease and death, with nearly half of cases genetically determined. Though genetic and high throughput sequencing-based approaches have identified sporadic and inherited mutations in a multitude of genes implicated in cardiomyopathy, how combinations of asymptomatic mutations lead to cardiac failure remains a mystery. Since a number of studies have implicated mutations of the transcription factor TBX20 in congenital heart diseases, we investigated the underlying mechanisms, using an unbiased systems-based screen to identify novel, cardiac-specific binding partners. We demonstrated that TBX20 physically and genetically interacts with the essential transcription factor CASZ1. This interaction is required for survival, as mice heterozygous for both *Tbx20* and *Casz1* die post-natally as a result of DCM. A *Tbx20* mutation associated with human familial DCM sterically interferes with the TBX20-CASZ1 interaction and provides a physical basis for how this human mutation disrupts normal cardiac function. Finally, we employed quantitative proteomic analyses to define the molecular pathways mis-regulated upon disruption of this novel complex. Collectively, our proteomic, biochemical, genetic, and structural studies suggest that the physical interaction between TBX20 and CASZ1 is required for cardiac homeostasis, and further, that reduction or loss of this critical interaction leads to DCM. This work provides strong evidence that DCM can be inherited through a digenic mechanism.

## Author summary

A molecular understanding of cardiomyocyte development is an essential goal for improving clinical approaches to CHD. While TBX20 is an essential transcription factor for heart development and its disease relevance is well established, many fundamental questions remain about the mechanism of TBX20 function. Principle among these is how *TBX20* mutations associated with adult dilated cardiomyopathy circumvent (DCM) the essential embryonic requirement for TBX20 in heart development. Here we report using an integrated approach that TBX20 complexes with the cardiac transcription factor CASZ1 in vivo. We confirmed TBX20 and CASZ1 interact biochemically and genetically, and show mice heterozygous for both *Tbx20* and *Casz1* die, beginning at 4 to 8 weeks post birth, exhibiting hallmarks of DCM. Interestingly, the human mutant TBX20^F256I^ bypasses the early essential requirement for TBX20 but leads to DCM. We report here that TBX20^F256I^ disrupts the TBX20-CASZ1 interaction, ascribing clinical relevance to this protein complex. Further, by using quantitative proteomics we have identified the molecular pathways altered in TBX20-CASZ1-mediated DCM. Together, these results identify a novel interaction between TBX20 and CASZ1 that is essential for maintaining cardiac homeostasis and imply that DCM can be inherited through a digenic mechanism.

## Introduction

Heart failure is a major cause of morbidity in the United States with more than 5 million people in the US living with this disease [1]. A major risk factor for developing heart failure is dilated cardiomyopathy (DCM). Clinically recognized as systolic dysfunction accompanied by dilation of one or both ventricles, DCM is a predominating cardiomyopathy and the most common disease requiring heart transplantation in the US [2, 3]; however, nearly half of DCM cases are of unknown etiology [4].

In efforts to understand the etiology of idiopathic DCM, mutations in over 50 genes including components of the contractile apparatus and cell cytoskeleton, as well as in factors involved in excitation-conduction coupling, have been identified as causative in DCM [5, 6]. However, few studies have explored the potential for aberrant transcriptional regulation of these factors to contribute to disease pathogenesis. In exception to this, recent studies have identified mutations in the T-box transcription factor *TBX20* associated with DCM [7–9].

Results of genetic analysis and protein depletion studies are consistent with an essential role for TBX20 during the early stages of vertebrate heart development [10–17]. Hearts lacking *Tbx20* show progressive loss of cardiomyocytes, failure of the heart to undergo looping and chamber formation, and defects in cardiomyocyte maturation [17–21]. In humans, loss-of-function mutations in *TBX20* can cause dilated cardiomyopathy, atrial septal defects, or mitral valve disease, while gain-of-function mutations in *TBX20* have been reported in patients with Tetralogy of Fallot (i.e., pulmonary outflow tract obstruction, ventricular septal defect, overriding aortic root and right ventricular hypertrophy) [7, 8, 22–24]. It has been further demonstrated that ablation of *Tbx20* in adult mouse cardiomyocytes leads to the onset of severe cardiomyopathy leading to death within 1–2 weeks after *Tbx20* loss [25].

While TBX20 is an essential transcription factor for heart development and its disease relevance is well established, many fundamental questions remain about the mechanism of TBX20 function. Principle among these is how *TBX20* mutations associated with DCM circumvent the essential embryonic cardiac requirement for TBX20.

To elucidate the mechanisms by which mutations in *TBX20* lead to human adult pathological states, we identified endogenous TBX20 cardiac protein-protein interactions by coupling a tagged endogenous allele of *Tbx20* with unbiased proteomic analysis. Results from these studies revealed TBX20 interacts with the essential cardiac transcription factor Castor (CASZ1), a gene that was also recently linked to DCM [26]. We confirmed that TBX20 and CASZ1 interact biochemically and genetically, and we go on to show that while mice singularly haploinsufficient for *Tbx20* or *Casz1* are asymptomatic, mice heterozygous for both *Tbx20* and *Casz1* die, beginning at 4 to 8 weeks post birth, and exhibit cardiomyocyte hypertrophy, interstitial fibrosis, and severe DCM. Interestingly, the human mutant TBX20^F256I^ bypasses the early essential requirement for TBX20 but leads to DCM. We report here that TBX20^F256I^ disrupts the TBX20-CASZ1 interaction, ascribing clinical relevance to this protein complex. Further, by using quantitative proteomics we have identified the molecular pathways altered in TBX20-CASZ1-mediated DCM. Together, these results identify a novel interaction between TBX20 and CASZ1 that is essential for maintaining cardiac homeostasis. These findings imply that DCM can be inherited through a digenic mechanism.

## Results

### Defining the endogenous cardiac TBX20 interactome

**Fig 1:**
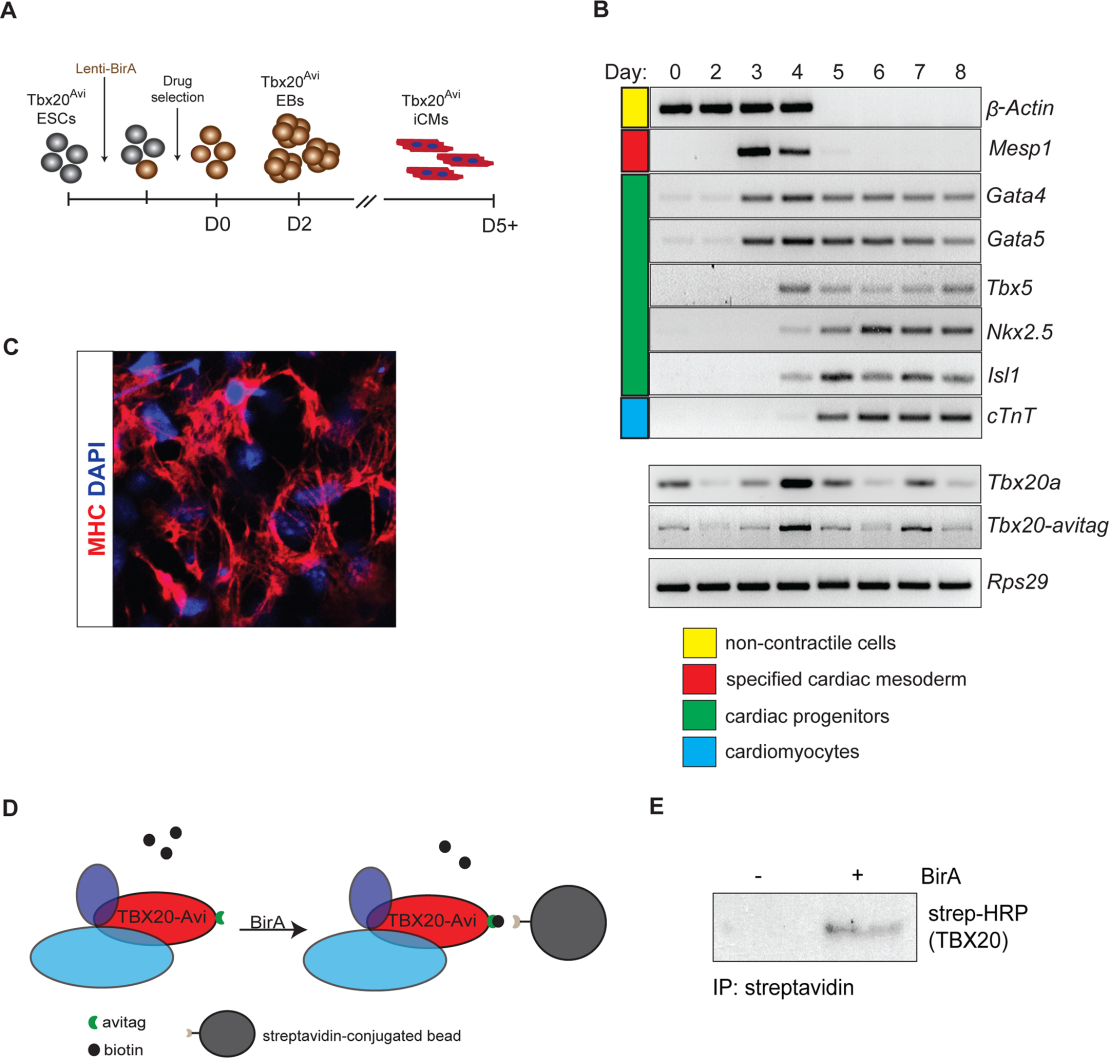
The TBX20^Avi^-BirA system for isolation of the TBX20 interactome. (A) Schematic diagram of ESC transduction with Lenti-BirA and subsequent differentiation. (B) RT-PCR panel showing changes in gene expression that ESCs undergo during 8-day differentiation into iCMs. (C) Immunohistochemistry of iCMs stained with cardiomyocyte marker Myosin heavy chain (MHC) and counterstained with DAPI. (D) Schematic of BirA-dependent biotinylation and streptavidin affinity isolation of TBX20 from iCMs. (E) Steptavidin affinity isolation of TBX20^Avi^ complexes confirmed in BirA-expressing iCMs by western blot analysis.

To identify endogenous protein interactions that regulate TBX20 function, we introduced the *Avitag*, in-frame, to the carboxy terminus of mouse *Tbx20* through homologous recombination in mouse embryonic stem cells (ESCs) (*Tbx20*^Avi^) (S1A and S1B Fig). Since the Avi-tag can be biotinylated through recognition of the Avi-tag sequence by the *E*. *coli* biotin ligase BirA [27, 28], we generated a lentivirus expressing BirA and transduced it into mouse *Tbx20*^*Avi/+*^ ESCs. After hygromycin selection, *Tbx20*^*Avi/+*^ ESCs that stably expressed BirA (S1C Fig) were differentiated into induced cardiomyocytes (iCM) using a serum-free differentiation method that routinely generates cultures containing >60% cardiomyocytes (Fig 1A) [29].

Expression analysis at each day of differentiation confirmed the *Tbx20*^*Avi*^:*BirA* ESCs recapitulated the wild-type cardiomyocyte differentiation program (Fig 1B). We further showed by Myosin Heavy Chain (MHC) expression and time lapse imaging that the *Tbx20*^*Avi*^:*BirA* ESCs differentiated into beating neonatal cardiomyocytes (Fig 1C, S1 Movie). Our analysis further verifies that *Tbx20*^*Avi*^ expression recapitulates endogenous *Tbx20* expression with highest levels in immature cardiomyocytes at day 4 and differentiated cardiomyocytes at day 7 (Fig 1B).

Published data has demonstrated a requirement for TBX20 in adult mice, with loss of TBX20 leading to abrupt cardiac failure [25]. Recently, mutations in *TBX20* in humans were associated with DCM [7–9]. To delineate the mechanisms of how *TBX20* DCM-associated mutations circumvent the essential requirements for TBX20 in cardiac development, we isolated and characterized the endogenous TBX20 cardiac protein interactome under physiological conditions from *Tbx20*^*Avi*^*; BirA* iCMs at day 7 of differentiation. As a control for non-specific interactions, identical affinity isolations were performed from BirA-negative iCMs. Proteins co-isolated from TBX20^Avi^ affinity purifications (APs) were analyzed by an SDS-PAGE tandem mass spectrometry-based proteomics approach, as in [30]. TBX20 was detected in the AP from BirA-expressing iCMs, with 19 unique tryptic peptides covering 54% of the TBX20 sequence (out of a theoretical maximum coverage of ~75%) (Fig 1D and 1E; S2A and S2B Fig; S1 Table).

**Fig 2:**
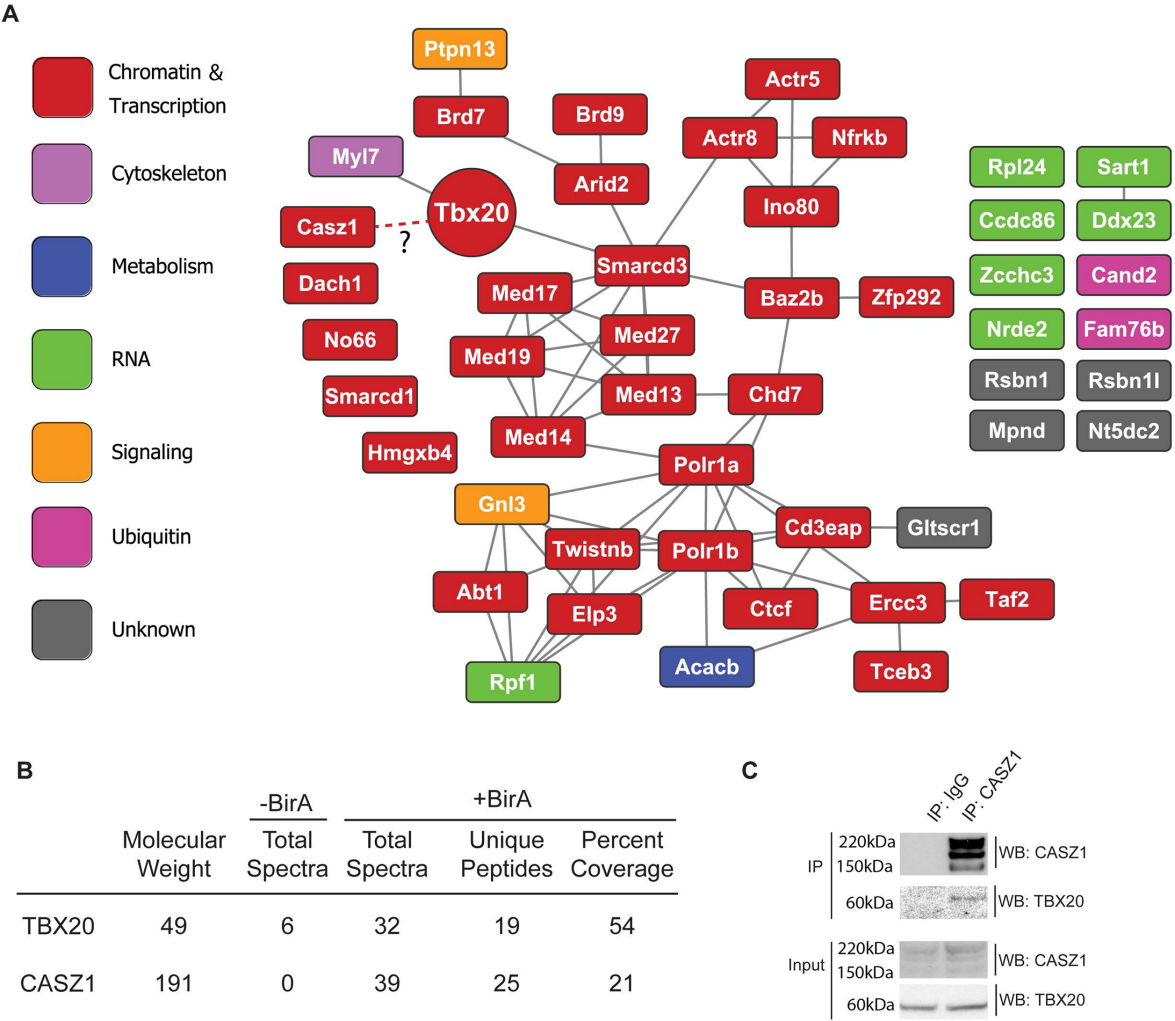
Endogenous TBX20 interactome. (A) Functional interaction network of the top 50 most enriched TBX20 interaction candidates. Known functional relationships are based on the STRING mouse database. Nodes are labeled with the protein’s gene symbol and color-coded based on its primary UniProt annotated cellular role. (B) Proteomic detection of TBX20 and CASZ1 in affinity purifications. The number of assigned spectra between control (-BirA) and BirA-expressing (+BirA) iCMs, unique peptides, percent amino acid sequence coverage, and respective molecular weights are provided. (C) Co-immunoprecipitations from *Xenopus laevis* embryos ectopically expressing full-length TBX20-EGFP alone, or in combination with full-length CASZ1-V5.

Identification of candidate high-confidence TBX20 interactions that have the potential to regulate cardiac functions was achieved using a multi-step bioinformatics approach based on the number of identified spectra per protein. First, interacting proteins identified by less than 10 spectra did not meet the identification requirement and were excluded from further analysis. Further, proteins identified in the *BirA*-expressing isolations were required to have at least a 4-fold increase in identified spectra over isolations from control iCMs. Due to the ascribed function of TBX20 as a critical cardiac transcription factor, we specifically focused on proteins with a nuclear or unknown subcellular localization. Finally, these interaction candidates were ranked by their AP enrichment (AP abundance versus whole cell abundance, S1 Table), which we have previously used to highlight the most prominent associations suitable for functional validation [31, 32]. Interestingly, the top 50 most enriched proteins in the TBX20 AP were predominately (32/50) annotated to Chromatin and Transcription gene ontologies (Fig 2A; S1 Table). Functional annotation of these proteins in the STRING database [33] revealed an interconnected network containing components of chromatin remodeling and RNA polymerase transcriptional complexes (including four components of the INO80 complex—Ino80, Actr5, Actr5, Nfrkb, and five components of the RNA Pol II mediator complex- Med13, Med14, Med17, Med19, Med27) (Fig 2A). These data suggest that TBX20 predominantly acts to regulate transcription in neonatal cardiomyocytes, likely via interactions with the INO80 and RNA Pol II mediator complexes.

### TBX20 complexes with CASZ1

In addition to identifying components of broadly expressed multiprotein chromatin machines, our analysis revealed the association of TBX20 with the essential cardiac transcription factor CASZ1 in BirA-expressing iCMs (25 unique peptides and 21% sequence coverage. As previously reported [34, 35], CASZ1 protein runs as three bands on at approximately 191kD, presumably due to post translational processing) (Fig 2B, S2C Fig). Surprisingly, this was the only developmentally-regulated cardiac transcription factor we found to interact with TBX20 in Day 7 cardiomyocytes. The low estimated cellular abundance of CASZ1 and the relatively high AP enrichment ratio (S1 Table) highlighted CASZ1 as a potential *in vivo* TBX20 interacting protein. We further confirmed the TBX20-CASZ1 interaction through reciprocal immuno-isolation of endogenous CASZ1 in cardiac nuclei from adult mouse hearts (Fig 2C) thus, verifying our ESC differentiation-based approach can successfully identify bona fide TBX20 interaction partners under physiological conditions. Since expression analysis and genetic fate mapping studies have shown CASZ1 is expressed only in cardiomyocytes and no other cardiac cell types [36], and since we were unable to identify this interaction at Day 4 of iCM differentiation, these studies imply the interaction between TBX20 and CASZ1 is temporally regulated and cardiomyocyte-specific.

Phylogenic analysis shows that TBX20 and CASZ1 are highly conserved across vertebrate orthologs [34, 35], suggesting that the TBX20-CASZ1 interaction may also be evolutionarily conserved. To confirm the interaction and to determine whether it is evolutionarily conserved, we injected *X*. *laevis* embryos with the *Xenopus* orthologous mRNAs of *TBX20* and *CASZ1*. In parallel, we co-expressed murine versions of tagged TBX20 and CASZ1 proteins in HEK293 cells. Immunoaffinity purification of TBX20 protein complexes from both of these sources, followed by immunoblotting confirms the formation of a TBX20-CASZ1 interaction in human cells and in *X*. *laevis* embryos (S3A and S3B Fig). Taken together, our findings are supportive of an evolutionarily conserved role for the formation of a TBX20-CASZ1 protein complex in differentiated cardiomyocytes.

### Combined haploinsufficiency of Tbx20 and Casz1 results in dilated cardiomyopathy

To determine the biological relevance of the TBX20-CASZ1 interaction, we tested for genetic interaction between *Tbx20* and *Casz1* by generating mice with cardiac-specific heterozygous loss of *Tbx20* and *Casz1* (*Tbx20*^*flox/+*^*; Casz1*^*flox/+*^*; Nkx2*.*5*^*Cre*^)[21, 36]. Compound heterozygous mice, hereafter referred to as *Tbx20*^*flox*/+^; *Casz1*^*flox/+*^, were born and appeared normal. However, beginning at 4 weeks of age we observed an increased incidence of death among *Tbx20*^*flox*/+^; *Casz1*^*flox/+*^ mice (2.7%) compared to the single heterozygotes (0%) (Table 1). This effect was amplified at later timepoints, with survival rates of 90.5% and 62.5% at 8 and 16 weeks of age, respectively, compared to a 100% survival rate in the single heterozygotes. Furthermore, we did not observe any overt phenotypes in *Tbx20*^*flox*/+^*; Nkx2*.*5*^*Cre*^ or *Casz1*^*flox/+*^*; Nkx2*.*5*^*Cre*^ mice. Since we were able to demonstrate that loss of *Casz1* does not affect *Tbx20* expression in adult heart tissue and that loss of *Tbx20* does not affect *Casz1* expression (S4A and S4B Fig), these studies are supportive of a genetic requirement for a functional interaction between TBX20 and CASZ1.

**Table 1:** Lethality in mutant and control mice at 4, 8, and 16 weeks old.

	Lethality*		
	4 weeks	8 weeks	16 weeks
***Nkx2*.*5*^*Cre*^**	0% (20)	0% (19)	0% (13)
***Tbx20*^*flox/+*^*; Nkx2*.*5*^*Cre*^**	0% (21)	0% (13)	0% (3)
***Casz1*^*flox/+*^*; Nkx2*.*5*^*Cre*^**	0% (13)	0% (7)	0% (2)
***Tbx20*^*flox/+*^*; Casz1*^*flox/+*^*; Nkx2*.*5*^*Cre*^**	2.7% (37)	9.5% (21)	37.5% (8)

*Lethality is expressed as a percentage of the total shown in parentheses.

### The TBX20-CASZ1 interaction is essential for normal cardiac function

**Fig 3:**
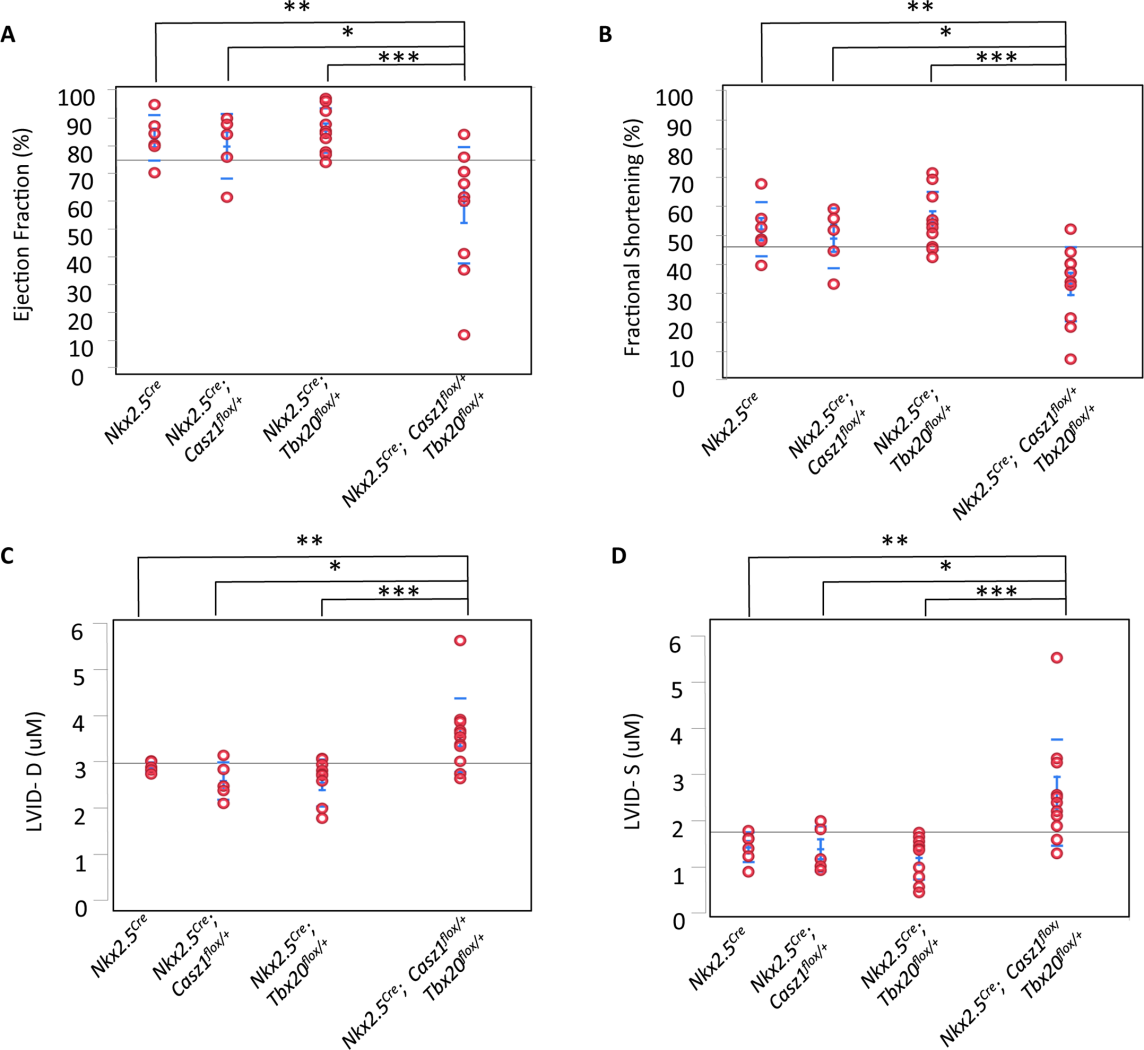
*Tbx20*^*flox/+*^*; Casz1*^*flox/+*^ compound heterozygotes display decreased cardiac function. (A-D) Echocardiography in mice aged 8–11 weeks, (A) left ventricular ejection fraction, (B) fractional shortening, (C) left ventricular inner diameter at diastole (LIVD-D), and (D) left ventricular inner diameter at systole (LIVD-S) was measured for mice in indicated cohorts. Each red circle represents one mouse analyzed within that cohort. *p<0.05, **p<0.005, ***p<0.0005. Statistical significance between pairs was calculated using Student’s t-test.

**Fig 4:**
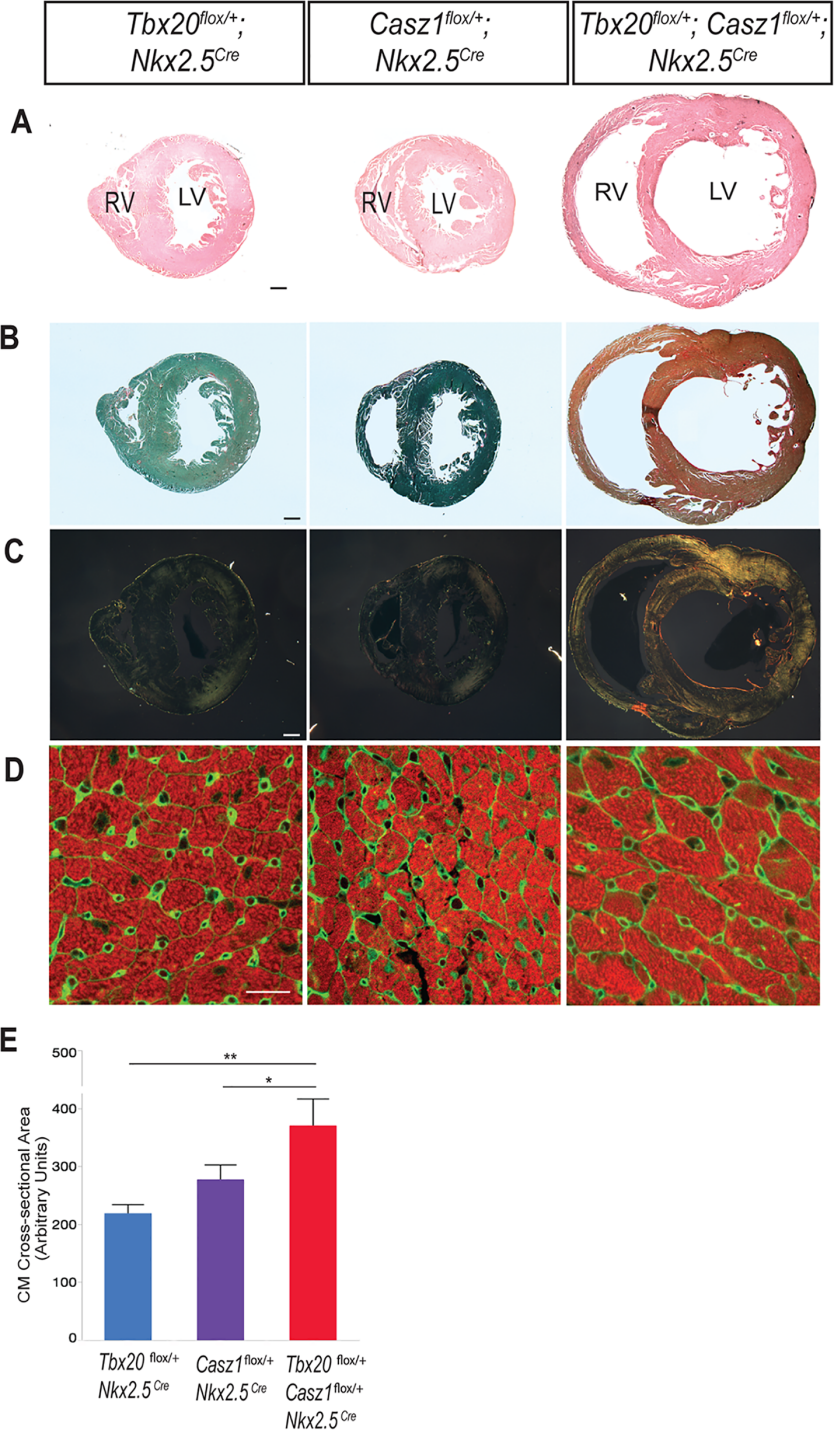
Double heterozygous hearts undergo pathological remodeling. (A-D) Hearts from mice aged 8–11 weeks. (A) Transverse sections of hearts stained with hematoxylin and counter-stained with eosin. (B) Transverse sections of hearts stained with Picrosirius red and fast green, and visualized using bright field microscopy. (C) Polarizing light microscopy of Picrosirius red-stained sections. Thin collagen fibers stain green to yellow, while thicker collagen fibers stain orange to red. (D) Transverse heart sections were immunostained with tropomyosin (red, cardiomyocytes) and WGA (green, cell membranes). Region of left ventricular free wall shown. (E) Quantification of cardiomyocyte cross-sectional areas, shown as mean ± SEM of 450+ cardiomyocytes per heart, n = 3 hearts per genotype. *p<0.05, **p<0.0005. Statistical significance between pairs was calculated using Student’s t-test. A-C, scale bar = 300 μm. D, scale bar = 20 μm.

To determine the cause of the reduced survival rate we observe in *Tbx20*^*flox*/+^; *Casz1*^*flox/+*^ mice, we performed detailed physiological analysis, using echocardiography, of single and compound heterozygous mice. These studies revealed that cardiac function is significantly compromised in *Tbx20; Casz1* compound heterozygotes compared to single heterozygotes. Compound heterozygotes exhibit significantly decreased ejection fraction and fractional shortening, increased left ventricular blood volume, and increased left ventricular diameter (Fig 3A–3D; Tables 2 and 3; S2 Table; S2–S6 Movies). Further, *Tbx20*^*flox*/+^; *Casz1*^*flox/+*^ heterozygous mice display dilated ventricles with a striking decrease in ventricular wall thickness compared to single heterozygotes (Fig 4A and 4B). These findings were observed in both male and female mice (S3 Table). Thus, *Tbx20*^*flox*/+^; *Casz1*^*flox/+*^ mice display defining anatomical features of DCM that progress to cardiac failure. Collectively, these findings suggest that the genetic interaction between *Tbx20* and *Casz1* is essential for normal cardiac homeostasis, and perturbation of this interaction leads to DCM.

**Table 2:** The TBX20-CASZ1 interaction is required for maintaining cardiac homeostasis in young adult mice (4–7 weeks old).

	*Casz1*^*flox/+*^*; Nkx2*.*5*^*Cre*^ (n = 6)	*Tbx20*^*flox/+*^*; Nkx2*.*5*^*Cre*^ (n = 8)	*Tbx20*^*flox/+*^*; Casz1*^*flox/+*^*; Nkx2*.*5*^*Cre*^ (n = 9)	ANOVA P Value
**LVEF (%)**	81.1 ± 2.7 *	88.2 ± 2.3 **	72.4 ± 2.2	0.0004
**LVFS (%)**	48.2 ± 2.9 *^,^ ^#^	57.6 ± 2.5 **	40.3 ± 2.3	0.0002
**LVVol;D (uL)**	25.9 ± 3.8	28.2 ± 3.3	27.5 ± 3.1	0.8951
**LVVol;S (uL)**	4.91 ± 1.3	3.68 ± 1.6	7.79 ± 1.1	0.0495
**LVID;D (mm)**	2.63 ± 0.15	2.72 ± 0.13	2.67 ± 0.13	0.9073
**LVID;S (mm)**	1.36 ± 0.13	1.17 ± 0.11	1.60 ± 0.11	0.0372
**IVS;D (mm)**	0.995 ± 0.06	0.937 ± 0.06	0.868 ± 0.05	0.3152
**IVS;S (mm)**	1.51 ± 0.07	1.54 ± 0.06 *	1.35 ± 0.06	0.0643
**LVPW;D (mm)**	1.14 ± 0.08	1.07 ± 0.07	1.05 ± 0.06	0.6722
**LVPW;S (mm)**	1.68 ± 0.06	1.69 ± 0.06	1.54 ± 0.05	0.1082

Mean ± SEM. LVEF- Left Ventricular Ejection Fraction; LVFS- Left Ventricular Fractional Shortening; LVVol;D- Left Ventricular Volume at end Diastole; LVVol;S- Left Ventricular Volume at end Systole; LVID;D- Left Ventricular Inner Diameter at end Diastole; LVID;S- Left Ventricular Inner Diameter at end Systole; IVS;D- Interventricular Septum thickness at end Diastole; IVS;S- Interventricular Septum thickness at end Systole; LVPW;D- Left Ventricular Posterior Wall at end Diastole; LVPW;S- Left Ventricular Posterior Wall at end Systole.

*p<0.05

**p<0.005 (indicated control groups were compared to compound heterozygotes)

^#^p<0.05 (indicated group was compared to *Tbx20*^*flox/+*^*; Nkx2*.*5*^*Cre*^).

**Table 3:** The TBX20-CASZ1 interaction is required for maintaining cardiac homeostasis in 8-11wk old mice.

	*Nkx2*.*5*^*Cre*^ (n = 6)	*Casz1*^*flox/+*^*; Nkx2*.*5*^*Cre*^ (n = 5)	*Tbx20*^*flox/+*^*; Nkx2*.*5*^*Cre*^ (n = 10)	*Tbx20*^*flox/+*^*; Casz1*^*flox/+*^*; Nkx2*.*5*^*Cre*^ (n = 11)	ANOVA P Value
**LVEF (%)**	82.2 ± 5.7 **	79.3 ± 6.3 *	84.8 ± 4.4 ***	58.8 ± 4.2	0.0010
**LVFS (%)**	50.6 ± 4.5 **	47.7 ± 4.9 *	53.5 ± 3.5 ***	31.8 ± 3.3	0.0006
**LVVol;D (uL)**	34.2 ± 8.7 *	27.5 ± 9.5 *	27.1 ± 6.7 **	58.9 ± 6.4	0.0078
**LVVol;S (uL)**	6.12 ± 8.8 *	6.24 ± 9.6 *	4.62 ± 6.8 *	30.0 ± 6.5	0.0403
**LVID;D (mm)**	2.97 ± 0.22 *	2.69 ± 0.25 **	2.65 ± 0.17 ***	3.63 ± 0.17	0.0017
**LVID;S (mm)**	1.47 ± 0.29 *	1.44 ± 0.32 *	1.26 ± 0.22 ***	2.55 ± 0.21	0.0012
**IVS;D (mm)**	0.90 ± 0.07	0.96 ± 0.08	1.01 ± 0.05 *	0.84 ± 0.05	0.1602
**IVS;S (mm)**	1.49 ± 0.09	1.49 ± 0.10	1.53 ± 0.07 *	1.25 ± 0.07	0.0457
**LVPW;D (mm)**	1.03 ± 0.11	1.17 ± 0.12	1.08 ± 0.08	1.00 ± 0.08	0.6740
**LVPW;S (mm)**	1.70 ± 0.11 *	1.72 ± 0.12 *	1.73 ± 0.08 **	1.36 ± 0.08	0.0138

Mean ± SEM. LVEF- Left Ventricular Ejection Fraction; LVFS- Left Ventricular Fractional Shortening; LVVol;D- Left Ventricular Volume at end Diastole; LVVol;S- Left Ventricular Volume at end Systole; LVID;D- Left Ventricular Inner Diameter at end Diastole; LVID;S- Left Ventricular Inner Diameter at end Systole; IVS;D- Interventricular Septum thickness at end Diastole; IVS;S- Interventricular Septum thickness at end Systole; LVPW;D- Left Ventricular Posterior Wall at end Diastole; LVPW;S- Left Ventricular Posterior Wall at end Systole.

*p<0.05

**p<0.005

***p<0.0005 (indicated control groups were compared to compound heterozygotes).

### Tbx20; Casz1 compound heterozygous hearts undergo cardiac remodeling

One of the defining clinical features of severe DCM is an accumulation of myocardial collagen leading to interstitial fibrosis, a contributing and compounding factor in cardiac dysfunction [37–39]. To confirm that the severe cardiac dysfunction we observe in *Tbx20; Casz1* compound heterozygotes is associated with advanced DCM, we examined collagen fibers and found robust collagen deposition in the interstitium of *Tbx20*^*flox*/+^; *Casz1*^*flox/+*^ hearts (Fig 4B and 4C). Despite the interstitial fibrosis and severely impaired systolic function, the fact that over half of these mice survive to adulthood with some degree of cardiac function led us to hypothesize that *Tbx20; Casz1* compound heterozygous cardiomyocytes undergo compensatory pathological hypertrophy. To test this hypothesis, we measured cardiomyocyte cross-sectional areas and found that MF20-positive cells in compound heterozygotes were indeed increased in size relative to controls (Fig 4D and 4E). This data suggests that disrupting the TBX20-CASZ1 interaction leads to severe DCM and cardiac fibrosis. In response to this heightened cardiac stress, *Tbx20; Casz1* compound heterozygote hearts appear to undergo pathological hypertrophy as an adaptive response.

### A human DCM-associated TBX20 mutation reduces the TBX20-CASZ1 interaction

**Fig 5:**
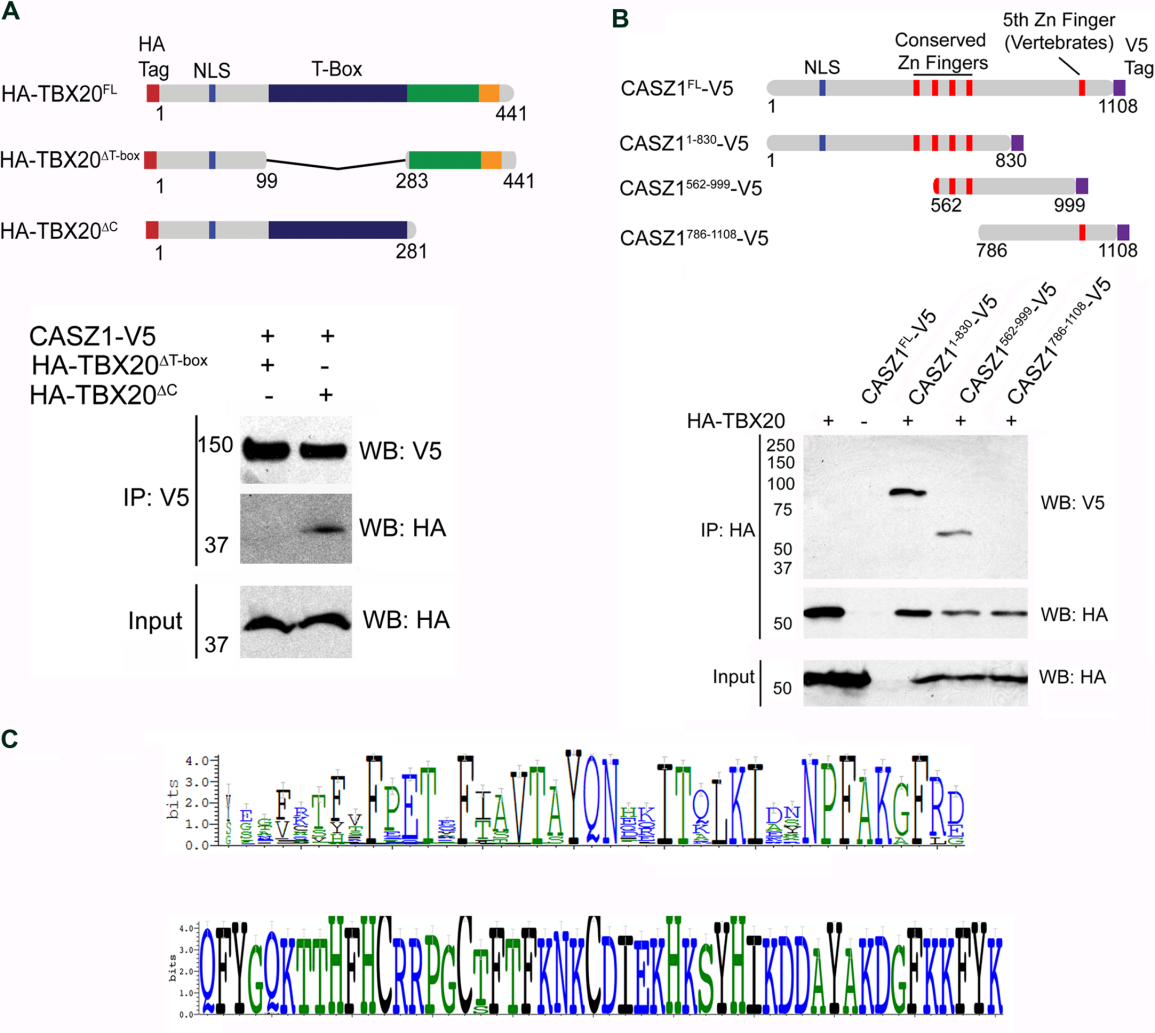
TBX20 and CASZ1 interact through their DNA binding domains. (A) (Top) Schematic of full-length TBX20 and truncations. NLS, Nuclear Localization Signal. Putative activation domain shown in green and putative repression domain in orange. (Bottom) Co-immunoisolations from *X*. *laevis* embryos expressing full-length CASZ1-V5 and either full-length HA-TBX20 or deletions shown in top panel. (B) (Top) Schematic of full-length CASZ1 and the truncations used in the co-immunoisolations. (Bottom) Co-immunoisolations from X. laevis embryos expressing full-length HA-TBX20 alone, or in combination with either full-length CASZ1-V5 or truncations shown in top panel. (C) (Top) Sequence alignment of TBX20 position 248–288 across 90 TBX20 orthologs. Height of letters is relative to conservation at that residue. (Bottom) Sequence alignment of CASZ1 at positions 601–650 across 90 CASZ1 orthologs.

Our data implies the TBX20-CASZ1 interaction is essential for normal cardiac homeostasis. To define the region of TBX20 that mediates interaction with CASZ1, we conducted immunoisolations with wild-type and deletion mutants in which either the T-Box or the C-terminus of TBX20 has been removed (Fig 5A). Immunopurifications of CASZ1 in the presence of wild-type TBX20, TBX20^ΔT-box^, or TBX20^ΔC^, show that the T-box domain is required for interaction with CASZ1, but that the C-terminus is dispensable (Fig 5A). In reciprocal studies, we find the four most amino-terminal zinc finger domains of CASZ1 are necessary for interaction with TBX20 (Fig 5B). The CASZ1-interacting region of TBX20, as well as the TBX20-interacting region of CASZ1, are highly conserved across species implying functional relevance to these regions (Fig 5C).

**Fig 6:**
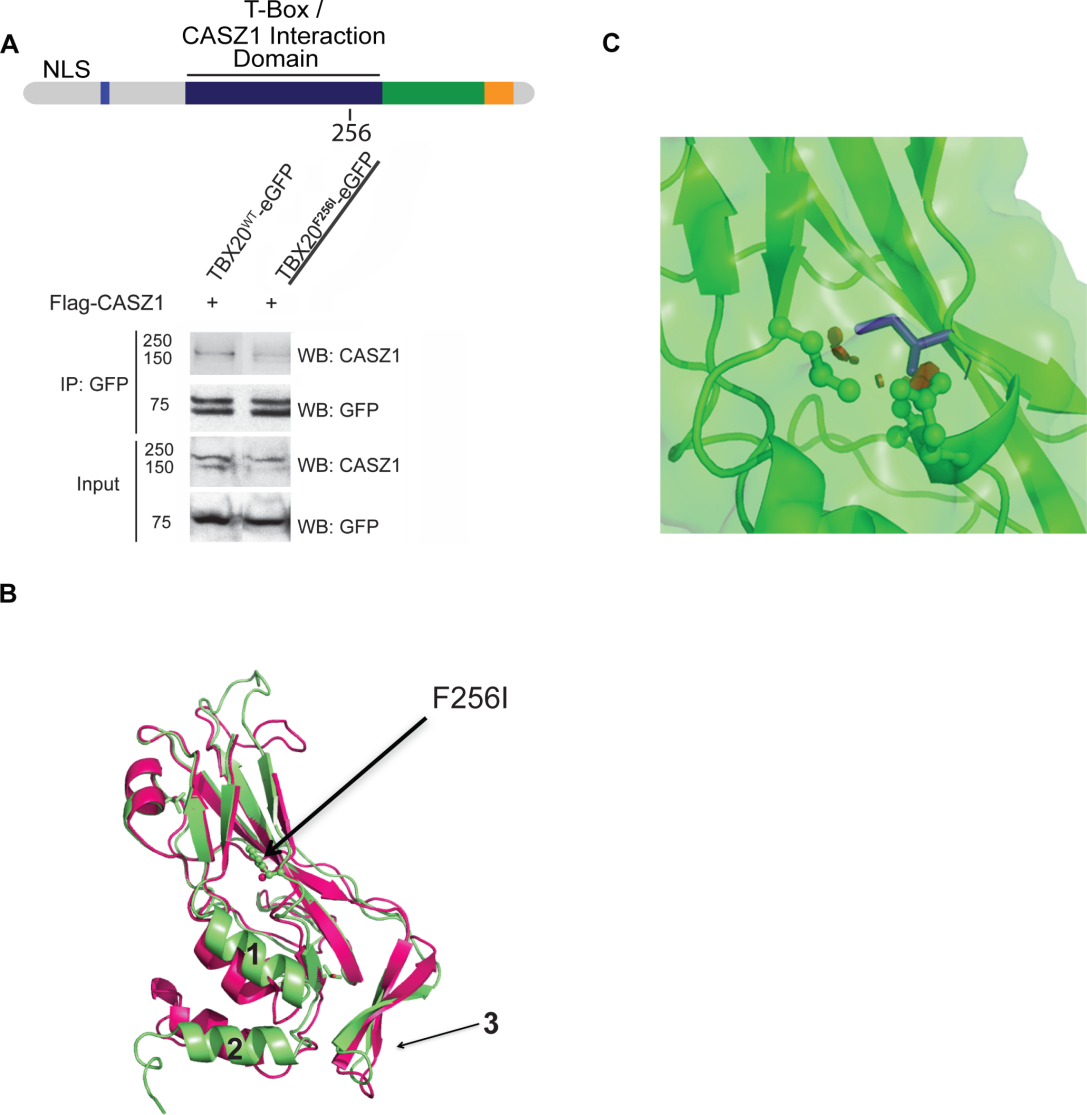
TBX20^F245I^ mutant displays impaired interaction with CASZ1. (A) (Top) Schematic of full-length TBX20 with location of *F256I* mutation shown. (Bottom) Co-immunoisolations of full-length wild-type CASZ1 with wild-type TBX20 or TBX20^F256I^. (B) Ribbon models of the average structures of TBX20 calculated from the 100 ns molecular dynamics simulations of the T-box domain. Left panel: starting structure of wild-type TBX20 (cyan) including DNA for reference. Right panel: an overlay of wild-type TBX20 (green) and TBX20^F256I^ (magenta). F256 and I256 side chains are displayed in stick form. Regions designated as 1, 2, and 3 undergo mutation-induced conformational changes in the unbound form. (C) Enlargement of the F256I residue shown in (B) in a different side chain rotamer that also induces steric clashes. The I256 mutation is rendered in purple stick form, with small red discs indicating steric clashes between the side chain of I256 and T-box residues E258 and T259.

Recently, human *TBX20* mutations have been identified that are associated with DCM; however, only one of these mutations, *TBX20*^*F256I*^, co-segregates in a dominant manner with complete penetrance in a family with DCM [9]. Moreover, DCM was found in all affected family members reported as healthy during health assessments performed when they were juveniles. The functional relevance of the *F256I* mutation is further underscored by the finding that the amino acid disrupted by this mutation is 100% conserved across all *TBX20* orthologs and by the observation that no *F256I* mutations were identified in 600 control samples [9]. Interestingly, the *F256I* mutation associated with DCM lies within the *TBX20* T-box domain, the region we found essential for interaction with CASZ1 (Figs 5A and 6A). To test if TBX20^F256I^ perturbs the TBX20-CASZ1 interaction, we performed immunoaffinity purifications of CASZ1 in the presence of wild-type TBX20 or TBX20^F256I^. Interestingly, the *F256I* mutation significantly reduces the interaction with CASZ1 (Fig 6A). These data imply that the DCM mutation *F256I* may contribute to the development of cardiac disease by disrupting a critical physical interaction between TBX20 and CASZ1.

### The TBX20^F256I^ mutation acts to sterically inhibit the TBX20-CASZ1 interaction

To gain a structural understanding of how the F256I mutation disrupts the TBX20-CASZ1 interaction, we conducted molecular modeling of the wild-type and TBX20^F256I^ T-box domain (Fig 5B and 5C). The predicted structures were based on the range of fluctuations in the structure that occur over a period of 100 ns (S4 Movie). Three regions are highlighted which show conformational changes induced by the mutation (Fig 6B). Our models find F256 is not predicted to contact DNA but the conversion of phenylalanine to isoleucine at position 256 leads to steric clashes with the conserved T-box residues E258 and T259 (Fig 6C). The critical functional nature of this region of TBX20 is underscored by the complete conservation of amino acids at residues F256, E258, and T259 across 250 members of the T-box gene family (S5A and S5B Fig). Taken together, these findings imply that F256I leads to a conformational change across the surface predicted to interact with CASZ1, and that disruption of this interaction leads to alteration in DNA binding.

To determine the transcriptional consequences of TBX20^F256I^ on the TBX20:CASZ1 interaction, we conducted transcriptional assays with TBX20, CASZ1 and TBX20^F256I^ alone and in combination. Results demonstrate TBX20 synergistically acts with CASZ1 and that TBX20^F256I^ significantly diminishes transcriptional activation by TBX20:CASZ1 (S6 Fig). These data together with our structural studies provide a mechanistic basis for how *F256I* disrupts TBX20:CASZ1 function.

### Tbx20; Casz1 compound heterozygous hearts have an altered DCM-associated proteome

To identify the molecular pathways altered in DCM haploinsufficient mutant (*Tbx20*^*flox/+*^*; Casz1*^*flox/+*^*; Nkx2-5*^*Cre*^) mouse hearts, we used quantitative multiplexed mass spectrometry to identify proteins with altered abundances relative to control hearts. Proteins were extracted from nuclear-enriched mouse cardiac fractions of mutant and control (*Nkx2-5*^*Cre*^) mice in duplicate and digested in-solution with trypsin. Peptides from each sample were labeled with different isobaric tandem mass tagging (TMT) reagents, pooled, fractionated, and analyzed by reverse phase nanoliquid chromatography coupled to a high resolution quadrupole Orbitrap tandem mass spectrometer. Using this strategy, 3164 proteins were identified and quantified based on their respective sequenced peptides and TMT reporter ions, respectively (S4 Table).

**Fig 7:**
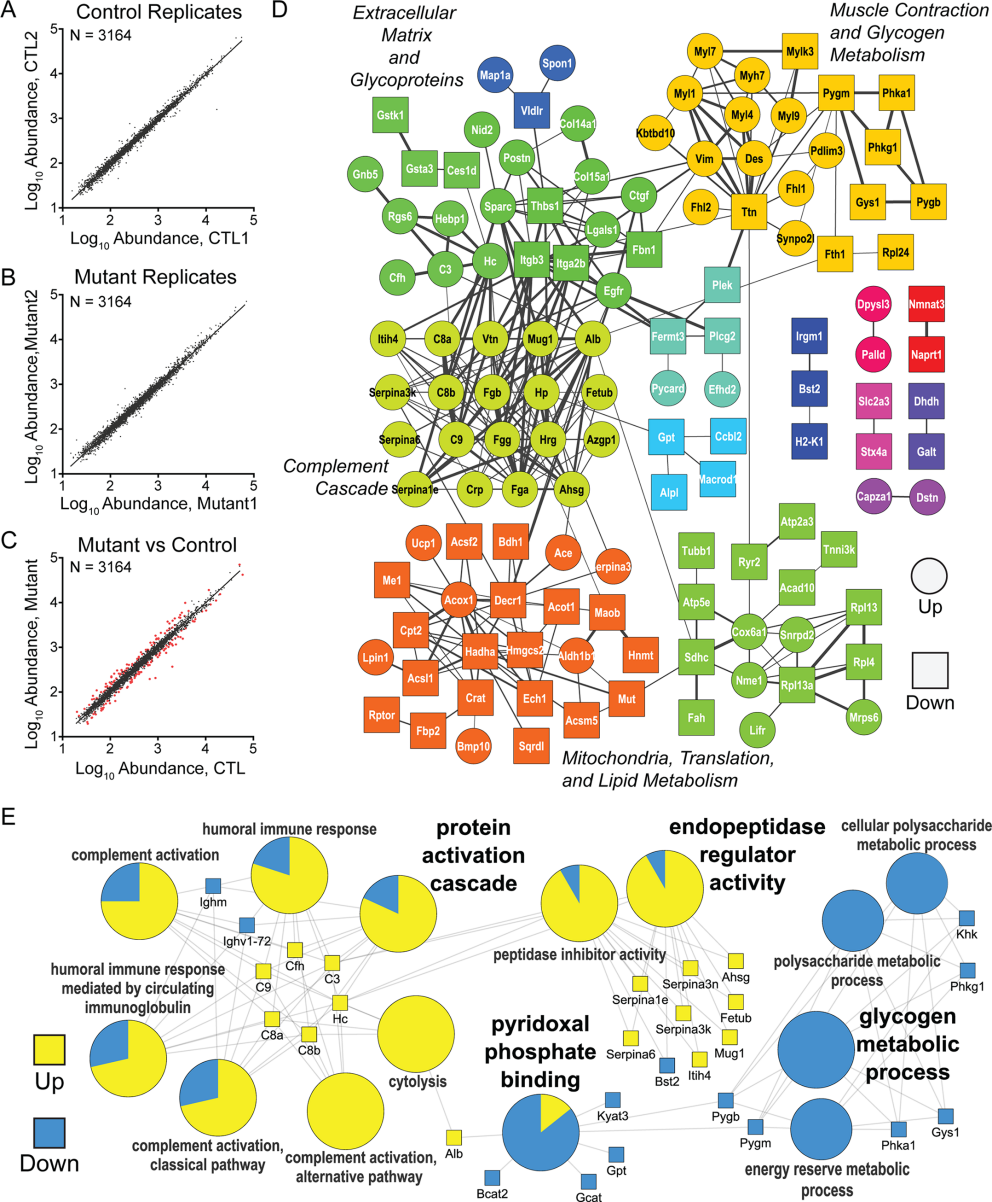
Proteomic analysis of DCM mouse hearts reveals activation of complement cascades and decreased protein abundance involved in glycogen metabolic processes. (A-B) Comparison of normalized protein abundances (black dots, n = 3164) quantified by TMT-based proteomics between biological replicates of (A) *Nkx2*.*5*^*Cre*^ (Control, CTL) and (B) *Tbx20*^*flox/+*^*; Casz1*^*flox/+*^*; Nkx2*.*5*^*Cre*^ (Mutant) mouse hearts. (C) Comparison of average normalized TMT protein abundances between Mutant and CTL mouse hearts (n = 2). Proteins classified as differentially abundant in the Mutant (TMT abundance ratios ≥ ±1/3-fold in both replicates) are indicated (red dots, n = 175). (D) Functional interaction network of differentially abundant proteins constructed using STRING interaction database. Proteins with up- and down-regulated abundances are represented by circle and square nodes, respectively, and labeled with primary gene symbols. Proteins that did not have connectivity to at least one other protein were excluded. Nodes are color-coded by cluster connectivity, which was assigned by the ReactomeF1 plug-in (see Methods). (See S7 Fig) Network edges reflect known STRING database relationships (confidence score >0.4). Edge thickness correlates with STRING confidence score (0.4–1). (E) Gene ontology (GO)-based network of selected over-represented Biological Processes represented by the differentially abundant proteins. Over-represented GO terms (p-value <0.05) are colored according to the proportion of the total differentially abundant proteins annotated to that term that were up- (yellow) or down- (blue) regulated. Protein to GO term assignments are indicated by network edges connecting the respective gene symbols (squares) with their assigned GO term.

To define the TMT ratio threshold for differential relative abundance, protein abundance values were compared between biological duplicates (S7 Fig). For both the control and mutant replicates, the correlation of abundances was high (R^2^ = 0.99) and the majority of proteins had low dispersion from a 1:1 linear curve (S7A and S7B Fig), indicating low biological and technical variation. Curve-fit analysis of TMT abundance ratio histograms for the control and mutant biological duplicates showed that, on average, 90% of the ratios varied less than ±30% (S7 Fig). Based on this result, a relative abundance ratio of at least ±1.3-fold between mutant and control mice in both replicates were used to identify a protein as differential. From the total number of quantified proteins, 175 met this criterion, of which 86 and 89 were up and down-regulated, respectively (Fig 7C; S4 Table). Further verifying the role of the TBX20; CASZ1 interaction, 165 of the 175 of the proteins identified by this approach were encoded by a gene previously demonstrated to be a TBX20 target [40](S5 Table).

To generate an initial picture of potentially dysregulated pathways, the known functional connectivity among differential proteins can be determined using databases of annotated pathways and protein-protein interaction. Towards this goal, known functional associations among the 175 differential proteins were scored based on the STRING bioinformatics database [41], and the relational networks visualized in Cytoscape [42] (Fig 7D; S8 Fig). A high degree of interconnectivity was observed among the differential proteins as 127 of the 175 annotated proteins had at least one other connection and each protein on average was connected to 4.6 neighbors. Network clustering was performed to identify subsets of highly connected proteins, which likely share similar functions. Overall, there are 10 functional clusters containing at least 3 proteins, indicated by the color-coding in Fig 6C. To identify the most significant biological processes and pathways that are perturbed in *Tbx20; Casz1* hypomorphic DCM hearts, we performed comparative Gene Ontology over-representation analysis of the differentially regulated proteins using ClueGO [43] (Fig 7E). Consistent with studies demonstrating an association between DCM and inflammation [44, 45], we found components of the pro-inflammatory response (i.e. complement activation) significantly up-regulated.

In addition to inflammation-associated proteins, our data further revealed a dysregulation of mitochondrial proteins known to be associated with impaired cardiomyocyte contractile function in DCM [46]. In line with these findings and the observation that reduced contractile force is linked to altered glycogen metabolism and cardiomyopathy [47–49], we found an over-representation of proteins associated with the glycogen metabolic pathway. We note that these were exclusively down-regulated proteins, represented by glycogen synthase (Gys1), glycogen phosphorylases (Pygm/Pygb), and phosphorylase kinase gamma 1 (Phkg1). Interestingly, proteins involved in glycogen regulation and in myosin-dependent muscle contractility were part of the same functional cluster (Fig 7D, yellow nodes); however, their individual abundances were down- and up-regulated, respectively (Fig 7D, circle vs. square nodes). Taken together, these data confirm at the protein level the DCM pathology in *Tbx20; Casz1* hypomorphic DCM mice.

### TBX20; CASZ1-linked DCM is associated with dysregulation of cell-cell adhesion proteins

In addition to proteins previously reported to be associated with DCM, our analysis identified a distinct set of cell-cell adhesion proteins in *Tbx20; Casz1* compound heterozygous hearts that were significantly overrepresented compared to whole genome annotation (Fig 7E; S8 Fig, yellow). These observations highlight the significant changes that are likely occurring in the extracellular and intracellular spaces and raise a key question- what are the signaling mediators that link these processes? To identify potential key mediators of TBX20-CASZ1-driven DCM, we constructed a gene-linked GO network for the Cellular Component ontology (S8 and S9 Figs). This network highlighted two interesting candidates, bone morphogenic protein 10 (Bmp10) and thrombospondin 1 (Thbs1), the former being a TGF-beta receptor ligand and the latter having roles in cell-cell adhesion as well as ER stress response [50]. Overall, this systems-level proteome view of DCM provides potential downstream targets and pathways that may be influenced as a result of *Tbx20* and *Casz1* haploinsufficiency and suggests a role for cell-cell adhesion in mediating DCM.

## Discussion

One in five adults free of cardiovascular disease by the age of 40 are at risk of developing congestive heart failure over their lifetime [51, 52]. Within this population DCM remains one of the leading causes of disease and death with nearly half of DCM cases genetically determined [53–57]. To date, most DCM mutations have been identified in genes coding for components of the contractile apparatus or the cell cytoskeleton or factors involved in excitation-conduction coupling. Though these studies have provided insight into the pathology of DCM, the transcriptional regulation of DCM is poorly understood. Recently, studies have identified mutations in the T-box transcription factor *TBX20* in DCM patients [7–9]. However, these studies have not explained how DCM-associated mutations bypass early essential requirements for *TBX20*. Here, we demonstrate TBX20 and CASZ1 physically and genetically interact in the adult heart and establish that this interaction is essential for cardiac homeostasis. We further find that disruption of the TBX20-CASZ1 interaction in mice and humans leads to cardiomyopathy. Mice singly heterozygous for alleles of *Tbx20* or *Casz1* are asymptomatic, while *Tbx20; Casz1* compound heterozygotes die post-natally, exhibiting systolic dysfunction, as well as ventricular dilation and interstitial fibrosis. These cardiac defects, in the absence of coronary artery disease or substantially abnormal load, are defining features of DCM as seen in human patients [6, 58, 59].

### CASZ1 and DCM

CASZ1 is a large para-zinc finger protein of unique structure and to date, there have been limited studies on the mechanisms of how CASZ1 regulates transcription [60–62]. These types of studies have been compromised by the lack of high-affinity high-specificity mammalian CASZ1 antibodies, precluding approaches such as ChIP-seq. It further remains unclear if CASZ1, as a para-zinc finger protein, directly binds DNA or is recruited via other transcription factors. Our structural studies favor a model by which the TBX20-CASZ1 interaction is required for DNA binding. This model predicts that the respective region of TBX20 that binds CASZ1 is near to or contributes to the DNA binding interface and has the potential to impact CASZ1 binding.

CASZ1 was first ascribed a role in vertebrate cardiovascular development in *Xenopus* [34, 62, 63]. Subsequent genetic studies in mammals uncovered that like TBX20, CASZ1 functions in the embryonic heart to control cardiomyocyte proliferation, with loss of CASZ1 leading to cardiac death by E12.5 [36, 64]. Our finding that *Tbx20; Casz1* compound heterozygous mice die post-natally implies that CASZ1 has a second and later role in cardiac homeostasis. This model is supported by the recent finding that mutations in *CASZ1*, like *TBX20*, are associated with human DCM [26].

### TBX20-CASZ1 protein pathways and DCM

In these studies, the *Nkx2*.*5-Cre* driver was used to generate mice null for *Casz1* and *Tbx20*. In all cases the *Nkx2*.*5-Cre* driver alone was used, with wild-type mice as a negative control in our physiological studies and in our quantitative proteomic studies. In contrast to previous reports [65], we could detect no significant changes in any cardiac function in *Nkx2*.*5-Cre* mice relative to wild-type mice. These finding may be due to genetic background, the sex on which the *Cre* driver was delivered to the offspring, or environmental variability as reported for other lines [66, 67]. Regardless of the reason for the variability, we did find the *Nkx2*.*5-Cre* driver had a high recombination efficiency reducing the levels of TBX20 and CASZ1 by half (S4 Table). Since, reducing CASZ1 expression by half had no detectable alteration in *Tbx20* expression and vice versa, our data suggests a biochemical and genetic interaction between TBX20 and CASZ1. Our data further indicates that disruption of this complex leads to DCM in mice and humans.

A previously published model of DCM, the phospholamban R9C transgenic mouse [68], has also been studied by proteomic analysis [69, 70]. This model exhibits impaired calcium regulation in cardiomyocytes, accompanied by decreased cardiac contractility and premature mortality [68]. The GO-associated proteome changes that we found in the haploinsufficient mice share similarities with the Phospholamban R9C mice. Specifically, both mouse models show up-regulation of actin-myosin cytoskeletal networks and down-regulation of mitochondria-associated proteins involved in fatty acid oxidation. Interestingly, proteomic analyses performed on ventricular tissues from human patients with inflammatory DCM had similar findings [71]. Yet some functional protein classes in our *Tbx20-CASZ1* haploinsufficient DCM mice were distinct, including an up-regulation of the complement system and greater coverage of down-regulated proteins in glycogen metabolic processes. While we found the *Tbx20-CASZ1* haploinsufficient mice have evidence of differential regulation in calcium-binding proteins, not surprisingly, the Phospholamban R9C mice have more pervasive effects on calcium-dependent signaling, such as involving ER stress responses, though it is possible that these distinctions may be due to differences in the progression of the fibrosis associated with DCM.

One of the hallmarks of DCM is altered cardiomyocyte force transduction that is frequently associated with alteration in the composition or functions of intercalated discs- a cardiac-specific structure at the contact site between cardiomyocytes [72, 73]. Here, we observed a significant mis-regulation of proteins involved in cell-cell adhesion in heart tissue from *Tbx20; Casz1* heterozygous mice. Moreover, these include three proteins which are encoded by genes that when mutated are causative to DCM- *TTN*, *DES*, and *PDLIM3*. Thus, our findings imply that the TBX20-CASZ1 complex acts, at least in part, to control the electrical and mechanical integration of neighboring cardiomyocytes.

### TBX20, CASZ1, and digenic inheritance of DCM

The observation that the *TBX20*^*F256I*^ mutation leads to a decreased association with CASZ1, along with the finding that patients heterozygous for a predicted *TBX20* null mutation (*TBX20*^*Q195X*^) [23] also display DCM suggest that the *TBX20*^*F256I*^ mutation may be acting in a haploinsufficient fashion. However, only two of the individuals within a single pedigree with the *TBX20*^*Q195X*^ mutation display DCM while other individuals display a wide range of cardiac abnormalities [23]. Moreover, we have screened the Exome Aggregation Consortium (ExAc) reference set and have identified four variants in *TBX20* in individuals that are asymptomatic. All variants lead to a premature stop codon in one of the *TBX20* alleles and all would be predicted to be functionally null (introduction of stop codons into exons 2, 4, 7, and 8)[74]. Together, these findings imply that the function of the TBX20-CASZ1 complex in DCM is not dose dependent. Alternatively, individuals harboring the *TBX20*^*F256I*^ mutation have a genetically sensitized background leading to a varying degree of penetrance that is determined by modifying genes that may be carried within the *CASZ1* pathway.

In cardiovascular disease, genetic mutations often result in varying degrees of penetrance, and in extreme examples, the presence of a disease-causing mutation can be asymptomatic [75–80]. These phenomena have often been explained by the action of genetic modifiers in which one gene mutation is causative to CHD and a second mutation modifies the effect of the first. However, more recent studies suggest an alternate or additional mechanism by which complete penetrance is achieved in human disease states by genetic variation at one or more loci [81]. In digenic inheritance, two genetic mutations are required for the clinical phenotype with either mutations alone being asymptomatic. Our findings provide an example of digenic inheritance in DCM and suggest that mutations in *TBX20* or *CASZ1* could lead to susceptibility to DCM but in many cases are not in themselves causative. We would envision these findings are not restricted to *TBX20* and *CASZ1* but rather are applicable to other genes and other forms of congenital heart disease (CHD) and DCM, and predict that genome sequencing of familial CHD will ultimately reveal a spectrum of additional CHD susceptibility alleles.

## Materials and methods

### Generation of Tbx20^Avi^ allele

The *Tbx20*^*Avi*^ allele was created by introducing the biotin acceptor peptide (Avi) targeting cassette, similar to our previous study [82], in-frame to the terminal exon of *Tbx20* in collaboration with the UNC Animal Models Core and the UNC BAC Core (Chapel Hill).

### Generation of Tbx20^Avi^; BirA cell line

The *Tbx20*^*Avi*^*; BirA cell line* was generated by targeting a sequence containing the *Avitag* followed by a *loxP*-flanked *neo* cassette into the stop codon of exon 8 of a *Tbx20a* genomic fragment derived from a 129 Sv genomic BAC library. The targeting construct was linearized and electroporated into mouse embryonic stem cells (ESCs) of E14TG2a.4 origin. Targeted ESCs were placed under 250 μg/mL G418 selection for 7–10 days and G418-resistant ESC clones (n = 384) were screened for homologous recombination by Southern blot analysis. Three ESC clones were correctly targeted, and one of these clones was subsequently used to derive the *Tbx20*^*Avi/+*^*; BirA* cell line. Briefly, *Tbx20*^*Avi/+*^ ESCs were grown to approximately 40% confluence and transduced with 5 MOI *Lenti-BirA* for 8 hrs. Twenty-four hours following transduction, cells were placed under 200 μg/mL hygromycin selection for 4–5 days. Hygro-resistant *Tbx20*^*Avi/+*^ cells were subsequently used for cardiomyocyte differentiations.

### ESC differentiation

*Tbx20*^*Avi/+*^*; BirA* ESCs were maintained on gelatin-coated dishes in a feeder-free culture system and differentiated [29] in serum-free (SF) media according to the Keller protocol. Briefly, ESCs were trypsinized and cultured at 75,000 cells/mL on uncoated petri dishes in SF medium without additional growth factors for 48 hrs. Two-day-old aggregated embryoid bodies (EBs) were dissociated and the cells reaggregated for 48 hr in SF medium containing 5 ng/mL human Activin A, 0.1 ng/mL human BMP4, and 5 ng/mL human VEGF (all growth factors purchased from R&D Systems). Four-day-old EBs were dissociated and 2 x 10^6^ cells were seeded into individual gelatin-coated wells of a 6-well dish in StemPro-34 SF medium (Invitrogen) supplemented with 2 mM L-glutamine, 1 mM ascorbic acid, 5 ng/mL human VEGF, 20 ng/mL human bFGF, and 50 ng/mL human FGF10 (R&D Systems). Cardiomyocyte monolayers were maintained in this media for 4–5 additional days with cells typically beginning to beat 2 days after seeding onto gelatin (total of 7–8 days of differentiation).

### Immunofluorescence

For immunofluorescence of cardiomyocytes, four-day-old ES cell-derived EBs were dissociated and seeded into 8-well chamber slides precoated with 0.1% gelatin. Induced cardiomyocytes were fixed on day 7 of differentiation in 4% paraformaldehyde for 20 min at room temperature, washed (3 x 1X PBS), permeabilized in 0.1% Triton X-100 in 1X PBS for 10 min, and blocked (10% fetal bovine serum [FBS], 0.1% Tween 20 in 1X PBS) for 30 min. Anti-myosin heavy chain (Abcam) was applied overnight, followed by PBS washes (3 x 1X PBS), and incubation with goat anti-mouse Alexa 546 (Invitrogen) for 1 hr. Cells were incubated in DAPI (200 ng/mL in ethanol) for 30 min and visualized by confocal microscopy on a Zeiss 710.

### Proteomic analysis of Tbx20 affinity purifications

Protein preparations, conjugation of magnetic beads and immunoaffinity purification and mass spectrometry were conducted as previously reported [82]. All results are from a minimum of two independent biological replicates. Briefly, immunoisolated proteins were resolved (~ 4 cm) by SDS-PAGE, and visualized by Coomassie blue. Each lane was subjected to in-gel digestion with trypsin and analyzed by nanoliquid chromatography coupled to tandem mass spectrometry as previously reported [83]. Tandem mass spectra were extracted by Proteome Discoverer (ThermoFisher Scientific, ver 1.4), and searched with the SEQUEST algorithm against a theoretical tryptic peptide database generated from the forward or reverse entries of the mouse UniProt-SwissProt protein sequence database (2013/08) and common contaminants (total of 43, 007 sequences). SEQUEST search results were analyzed by Scaffold (version 4.6.1, Proteome Software Inc) using the LFDR scoring scheme to calculate peptide and protein probabilities. Peptide and protein probabilities thresholds were selected to achieve ≤ 1% FDR at the peptide level based on LFDR modeling and at the protein level, based on the number of proteins identified as hits to the reverse database. The spectral counts assigned to proteins that satisfy these criteria and had a minimum of two unique peptides were exported to Excel for data processing.

### Interaction bioinformatics analysis

Proteins identified by LC-tandem MS were filtered to exclude non-specific associations. Proteins were retained as specific interaction candidates if the proteins were assigned (1) at least ten spectral counts in the *Tbx20*^*Avi*^*;BirA* condition, and (2) were uniquely identified or had at least a 4-fold spectral count enrichment in the *Tbx20*^*Avi*^*; BirA* condition versus the control. Next, the subset of candidates assigned a nuclear or unknown UniProt subcellular localization were retained for calculation of enrichment index values, as previously described [31]. Briefly, the relative protein abundance within the affinity purification was calculated using the NSAF approach [84], then normalized by each protein’s respective cellular abundance estimated in the PAX database [85] (Mouse—whole organism, SC GPM 2014). Interaction candidates were ranked by their enrichment index and the top 50 proteins were analyzed by STRING [86] for interaction network analysis. Interactions with a combined STRING score of > 0.4 (medium confidence) were retained, exported, and visualized in Cytoscape (ver. 3.3). Proteins within the network were assigned into broad protein functional classes based on annotations in the UniProtKB database.

### Western blot analysis

Western blots were probed with the following primary antibodies overnight at 4°C: mouse anti-V5 (Invitrogen) 1:5000; mouse anti-GFP (JL-8, Clontech) 1:10000; mouse anti-HA-HRP (Cell Signaling #2999) 1:1000, mouse anti-GAPDH (Millipore) 1:1000, goat anti-TBX20 (Santa Cruz Biotechnology) 1:600, rabbit anti-CASZ (Santa Cruz Biotechnology) 1:1000, and chick anti-BirA (Abcam) 1:2000. After being rinsed, blots were rinsed in the following secondary antibodies for 1 hr at room temperature: anti-IgG2a-HRP (Jackson Immunoresearch) 1:10000. Antibody-antigen complexes were visualized using an ECL Western Blotting Analysis System (Amersham).

### Mice

*Tbx20*^*flox/+*^ mice were generously provided by Sylvia Evans (UCSD) [21]. The *Casz1*^*flox/+*^ mouse has been previously reported [36]. Histological sectioning and immunohistochemistry were done as reported except as noted [36]. All mice are on a mixed B6/129/SvEv/CD-1 background and all mouse experiments were performed according to the Animal Care Committee at the University of North Carolina, Chapel Hill.

### Echocardiography

Cardiac function was assessed in conscious 8–11 week-old *Casz1*^*flox/+*^*; Nkx2*.*5*^*Cre*^, *Tbx20*^*flox/+*^*; Nkx2*.*5*^*Cre*^, and *Tbx20*^*flox/+*^*; Casz1*^*flox/+*^*; Nkx2*.*5*^*Cre*^ mice (5–10 mice per genotype) by thoracic echocardiography using VisualSonics Vevo 770 ultrasound system (Visual Sonics, Inc.). All imaging was done by trained technicians blinded to the genotypes of the animals. Briefly, a topical hair removal agent was used on the chest and abdomen of mice. The mice placed on a warmed table in the supine position for imaging. A 30 MHz pediatric probe used to capture 2-dimensional guided M-mode views of the long and short axes at the level of the papillary muscle. VisualSonics Analytic software was used to determine mean ventricular wall and interventricular septum thickness, as well as the left ventricle diameter from at least 3 consecutive cardiac cycles. Means were used to calculate ejection fraction and fractional shortening.

### Statistics

All statistical analysis performed using SAS JMP 10. Statistical significance between individual groups was calculated using Student’s T-test, while significance between more than 2 groups was calculated using ANOVA.

## Supporting information

S1 Fig Construction of the Tbx20^Avi^ allele. (PSD)

S2 Fig MS/MS identification of TBX20 and CASZ1. (PSD)

S3 Fig The TBX20-CASZ1 interaction occurs with both Xenopus laevis and Mus musculus protein isoforms. (PSD)

S4 Fig TBX20 and CASZ1 expression in the left ventricle of control and double heterozygote hearts. (PSD)

S5 Fig TBX20 is highly conserved at F256, E258, and T259. (PSD)

S6 Fig Transcriptional assay demonstrating the reduction in ANF reporter activity due to the combined effects of TBX20 F256I and CASZ1. (PSD)

S7 Fig Distribution of TMT ratios for biological duplicates. (PSD)

S8 Fig STRING network of differential proteins coded by Mut/CTL TMT abundance ratio. (PSD)

S9 Fig Over-represented GO biological process network for differential proteins. (PSD)

S10 Fig Over-represented GO cellular component network for differential proteins. (PSD)

S1 Table Tbx20 interactions: MS/MS raw data. (XLSX)

S2 Table Cardiovascular physiology in young adult female and male mice. (AI)

S3 Table Cardiovascular physiology in mature adult female and male mice. (AI)

S4 Table TMT analysis: MS/MS raw data. (XLSX)

S5 Table Proteins differentially abundant in mutant hearts that are also TBX20 target genes by ChIP. (XLSX)

S1 Movie Day 7 iCMs. (MP4)

S2 Movie 11wk female *Nkx2*.*5* ^*Cre*^. (MOV)

S3 Movie 10wk female *Tbx20*^*flox/+*^*; Nkx2*.*5*^*Cre*^. (MOV)

S4 Movie 10wk female *Casz1*^*flox/+*^*; Nkx2*.*5*^*Cre*^. (MOV)

S5 Movie 11wk female double heterozygous. (MOV)

S6 Movie 11wk female double heterozygous. (MOV)
